# Research on the Development of Rural Human Settlement Environment in the Upper Reaches of the Lancang River Based on Ecological Carrying Capacity Constraints

**DOI:** 10.1002/gch2.202400248

**Published:** 2025-01-20

**Authors:** Xiaoliang Zhao, Junhuan Li

**Affiliations:** ^1^ School of Architecture Xi'an University of Architecture and Technology Xi'an 710055 P. R. China

**Keywords:** construction strategy, ecological carrying capacity, human settlements, suitability evaluation, the Sanjiangyuan region

## Abstract

The development of rural human settlement environments in ecologically sensitive areas, particularly the upper reaches of the Lancang River within the Sanjiangyuan region, has garnered significant attention due to its paramount importance in balancing economic growth and ecological conservation. However, the comprehension of sustainable development strategies within the confines of ecological carrying capacity in these regions remains inadequate. The present study aims to investigate the rural human settlement environment in Nangqen County, situated within the Sanjiangyuan region, and to explore adaptive construction strategies that facilitate harmonious coexistence between human settlements and the natural environment. Utilizing an ecological footprint model integrated with the ArcGIS platform, this research evaluates the ecological carrying capacity and its variations from 2000 to 2020. The results indicate that, despite the land ecological environment being generally safe, the total ecological footprint has surpassed a twofold increase, primarily driven by the expansion of animal husbandry activities. Furthermore, the study conducts a suitability assessment of rural settlements in Nangqen County, pinpointing areas that are most conducive to sustainable development and those that necessitate protection.

## Graphical abstract

This article systematically evaluates the rural human settlement environment in Nangqen County, upper reaches of the Lancang River, considering ecological carrying capacity constraints. Utilizing an ecological footprint model with ArcGIS, the study analyzes changes in ecological footprint, carrying capacity, and pressure from 2000–2020. Suitability assessment of rural settlements identifies areas for sustainable development and protection, proposing strategies to optimize settlement patterns and ensure ecological sustainability.

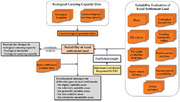

## 1 Introduction

In the expansive domain of human settlement research, the evolution of rural habitats continues to be a pivotal area of focus, highlighting the complex interplay between societal progress and environmental sustainability.^[^ ^1^, ^2^ ^]^ The historical progression from rudimentary settlements to sophisticated rural communities mirrors humanity's growing comprehension and adaptation to natural ecosystems.^[^ ^3^, ^4^ ^]^ This equilibrium between economic growth and ecological preservation is particularly crucial in regions like the Upper Reaches of the Lancang River, where delicate ecosystems and diverse biota coexist with rapidly developing rural areas.^[^ ^5^, ^6^, ^7^ ^]^ Within this context, the concept of ecological carrying capacity plays a critical role in guiding rural development.

While the field of rural development has witnessed notable emphasis on achieving sustainability within ecological carrying capacity, significant knowledge gaps persist,^[^ ^8^, ^9^ ^]^ especially concerning the specific challenges and opportunities facing regions such as the Upper Reaches of the Lancang River.^[^ ^10^ ^]^ Despite the ecological importance and distinct socio‐economic traits of this region, studies that integrate ecological carrying capacity with rural settlement development are scarce. This gap is particularly critical given the escalating pressures on the region's fragile ecosystems and rich biodiversity from rapid development.^[^ ^11^, ^12^ ^]^ Most existing research offers general analyses, often neglecting strategies tailored to specific regional needs that balance ecological and community growth.^[^ ^13^, ^14^, ^15^ ^]^

In the fields of protection and development of traditional villages, evaluation of ecological carrying capacity and adaptive construction, domestic and international studies have yielded significant findings. Research on highland rural settlements in the Sanjiangyuan region is still in its nascent stages, primarily focused on three key areas. The first major area of focus is the ecological environment and natural elements,^[^ ^16^, ^17^, ^18^, ^19^ ^]^ which accounts for over 70% of the most significant literature in this field. This reflects the current academic research's primary concentration on nature, ecology, and environmental science.^[^ ^20^ ^]^ The second key area is the study of environmental sustainability, exploring the interactions between human activities and the natural ecological environment.^[^ ^21^, ^22^ ^]^ The third area concerns the relationship between human living spaces and the natural environment, with a primary focus on the behavioral characteristics of human activities and related strategies,^[^ ^23^, ^24^, ^25^, ^26^ ^]^ while studies on the mechanisms of their interactions remain relatively limited.

The current state of research on the upper reaches of the Lancang River is constrained by three key limitations.^[^ ^27^, ^28^ ^]^ First, there is a paucity of in‐depth exploration into the formation and historical evolution of the settlement space.^[^ ^29^, ^30^ ^]^ Second, there is a notable absence of attention paid to the social structure and behaviour of the residents. The patterns of the residents in the region, especially the relationship between different groups and their locations in the villages, have not been sufficiently explored. Furthermore, due to the limited scope of the space, the research objects are usually individual villages and dwellings,^[^ ^31^, ^32^, ^33^ ^]^ which lack comprehensiveness and diversity. Therefore, in the follow‐up research work, it is necessary to combine the local natural environment and social and economic development status.

The protection and utilization of rural settlements, particularly those of the Tibetan people, are gradually evolving toward a new stage of systematization and refinement.^[^ ^20^ ^]^ This process entails a transition from the isolated safeguarding of individual edifices to a comprehensive examination of settlement spatial configuration,^[^ ^34^ ^]^ ethnic characteristics,^[^ ^5^ ^]^ architectural layout,^[^ ^21^ ^]^ and cultural ecology.^[^ ^35^ ^]^

The present study aims to fill this gap by examining the relationship between ecological limits and settlement patterns in this under‐researched region. It seeks to develop a framework for rural human settlement environments in the Upper Reaches of the Lancang River, with a specific focus on the constraints of ecological carrying capacity. Employing a multidisciplinary approach that integrates ecological footprint analysis, remote sensing technology, and GIS spatial analysis, this study investigates the development of rural human settlement environments in the biodiverse Upper Reaches of the Lancang River. By exploring the interaction between ecological boundaries and rural development dynamics, this research endeavors to provide a comprehensive understanding of how rural communities can thrive while adhering to the ecological constraints of their surroundings.

## 2 Experimental Section

### 2.1 Study Area

**Figure 1:**
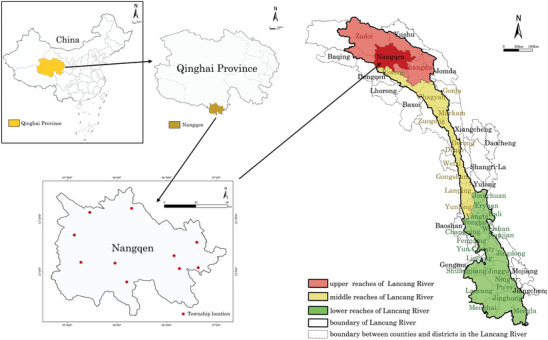
Geographical location of the region.

This study employs a comprehensive approach to assess the human living environment in the upper reaches of the Lancang River basin, with a particular focus on its geomorphological features for the purpose of ecological evaluation. The study area encompasses Zaduo County and extends to Qamdo, following established regional classifications to ensure administrative coherence and facilitate robust data collection.^[^ ^36^ ^]^ Nangqen County, situated on the eastern Qinghai‐Tibet Plateau, has been selected as the primary site due to its ecological significance in the Tibetan Kangba region, which is characterized by high population density and diverse topography (**Figure** **1**). The area is characterized by a diverse topography, with 51.2% of the landscape comprising mountains, 18.7% hills, and 30.1% plains. The region experiences alpine climates and a range of challenging conditions, including soil salinisation and erosion. Notwithstanding these challenges, the region's rich ecological diversity, including forests and wetlands, plays a pivotal role in biodiversity conservation and offers a distinctive setting for examining rural development models that strike a balance between economic growth and environmental protection. The county's agricultural and animal husbandry activities contribute to food security and economic stability,^[^ ^37^ ^]^ while its brine salt resources underscore the importance of geological diversity and resource management strategies for sustainable development.

### 2.2 Data Collection

The research places an emphasis on the assessment of ecological footprints in Nangqen County, utilising socioeconomic statistics and benchmarks from the World Wide Fund for Nature and the Food and Agriculture Organization of the United Nations. The core of the analysis is the examination of patterns of biological resource and energy consumption, with a particular focus on the region's economic structure. The ecological carrying capacity, including forest cover and agricultural land reclamation, was evaluated via remote sensing to assess forest growth, canopy density, and grassland quality.^[^ ^38^ ^]^ Furthermore, land use/cover was classified from 2000 to 2020 using Landsat‐TM/ETM imagery processed with ERDAS, ENVI, and ArcGIS, thereby ensuring precise categorization into the following land use categories: farmland, forest land, grassland, water, and construction land. **Table** **1** provides a comprehensive outline of the data sources used in this study.

**Table 1:** Data source summary.

Data	Source		Uses
SRTM DEM	https://www.gscloud.cn	30 m × 30 m/2000,2005,2009,2015,2020	For generating maps of slope, geological disaster risk, altitude, agricultural and animal husbandry radius, per capita production land area, public service facilities, water source distance, transportation accessibility, and urban radiation.
Landsat‐TM/ETM imagery	https://earthexplorer.usgs.gov/	30 m × 30 m/2000,2005,2009,2015,2020	For generating rural land suitability map
Life consumption data and demographic data	the Qinghai Statistical Yearbook, Yushu Prefecture Annals, and China Rural and Urban Statistical Yearbook	2000,2005,2009,2015,2020	For calculating the ecological footprint
Village location data	https://www.stats.gov.cn/	2020	For generating map of suitability evaluation for rural settlement land

SRTM = Shuttle Radar Topography Mission, DEM = Digital Elevation Model.

### 2.3 Thematic Layer Preparation

**Figure 2:**
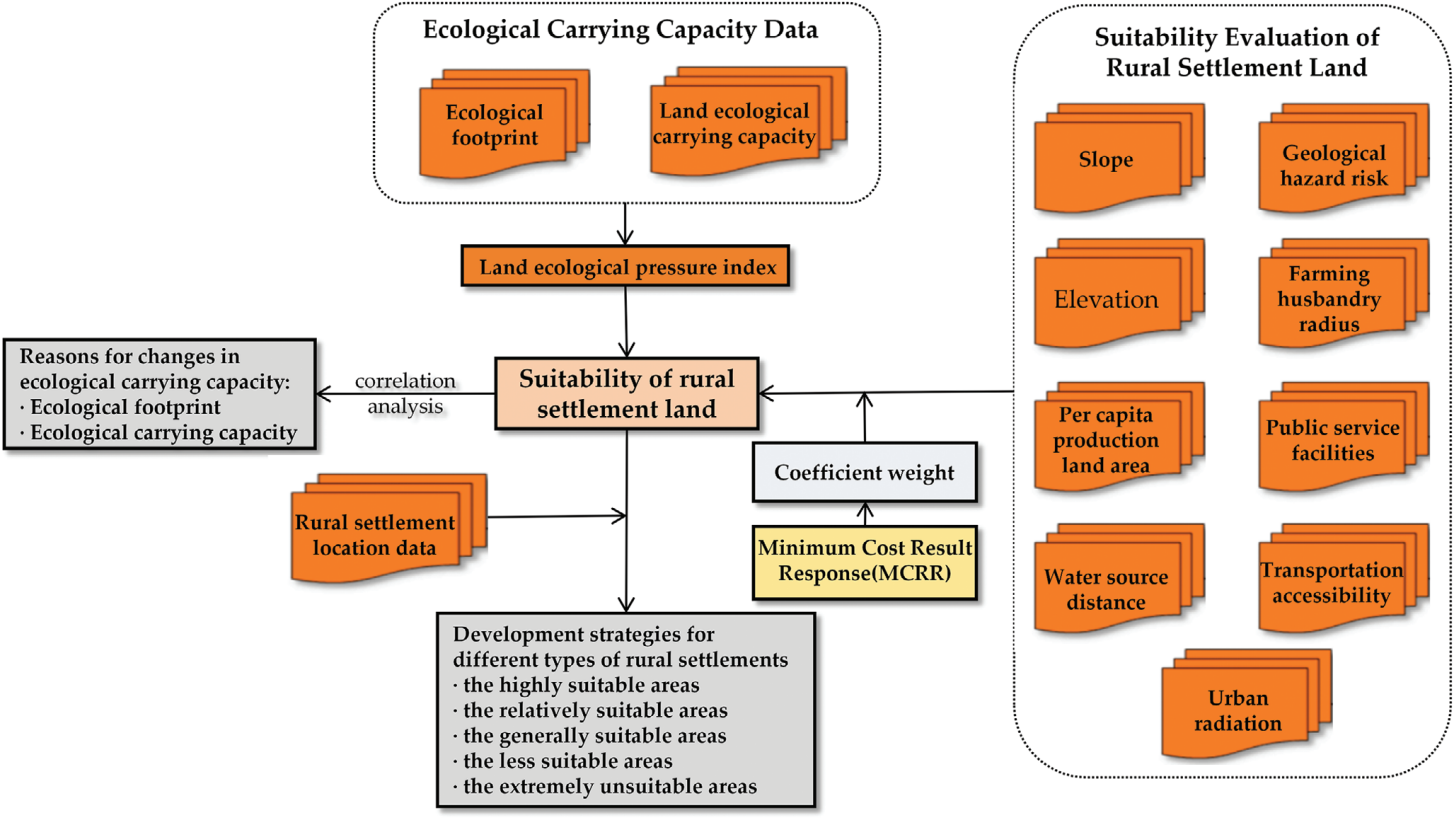
The process framework in this study.

This study employs the ecological footprint method to assess the ecological carrying capacity of Nangqen County. This is achieved by examining three indicators: ecological footprint, ecological carrying capacity, and ecological pressure. This approach allows for an evaluation of the development trajectory of local ecological carrying capacity. Subsequently, a suitability evaluation system for rural settlements was constructed to assess the current situation of local rural site selection. Based on this, the sustainable development of rural settlements in Nangqen County was studied in conjunction with the results of ecological carrying capacity. The study's overall framework is presented in **Figure** **2**.

By measuring indicators such as ecological footprint and ecological pressure in Nangqen County, it is possible to ascertain whether the production and consumption patterns of residents in the area are sustainable within the region's ecological carrying capacity. This is essential to achieve the objective of promoting harmonious and sustainable rural living and human living environments in the area. The specific Equation (1) for ecological footprint is as follows:^[^ ^39^ ^]^

(1) $$\mathrm{EF}=N\times \mathrm{ef}=N\times r_{j}\times \sum_{i=1}^{a}\mathrm{aai}=N\times \mathrm{rj}\times \sum_{i=1}^{a}\left(\frac{\mathrm{Ci}}{\mathrm{Pi}}\right)$$

In the above equations, EF represents the total ecological footprint; N is the population of the study area; ef is the per capita of ecological footprint; i is the type of consumer goods; j is the type of ecological productive land; aa_i_ is the per capita of ecological productive land area of the i‐th commodity; c_i_ represents the per capita of consumption of the i‐th commodity; p_i_ is the world's average annual production capacity of the i‐th commodity; r_j_ is the equilibrium factor of land type j.

#### 2.3.1 Land Ecological Capacity

The specific Equation (2) of land ecological carrying capacity is as follows:^[^ ^39^ ^]^

(2) $$\mathrm{EC}=N\times \mathrm{ec}=N\sum_{j=1}^{6}\mathrm{aj}\times \mathrm{rj}\times \mathrm{Yj}$$

In the above equations, EC represents the total ecological carrying capacity; N is the population of the study area; *ec* is the per capita of ecological carrying capacity; a_j_ is the per capita of ecological productive land area; r_j_ is the equilibrium factor of class j land; Y_j_ is the yield factor of class j land (**Table** **2**).

**Table 2:** Various coefficients of land.^[^ ^40^ ^]^

Land Type	The Yield Factor of Land	The Equilibrium Factor of Land
Farmland	0.46	2.21
Forest land	0.50	0.49
Grassland	1.13	1.34
Waters	1.13	0.36
Fossil energy land	0.00	2.21
Building land	0.00	1.30

#### 2.3.2 Land Ecological Pressure Index

The Land Ecological Pressure Index (*LEPI*) is defined as the quotient of the ecological footprint and the ecological carrying capacity within the designated study area.^[^ ^41^ ^]^ This ratio elucidates the interplay between the regional ecological demand and the ecological capacity.^[^ ^42^ ^]^ The *LEPI* serves as a metric to quantify the magnitude of anthropogenic impact on the ecological environment.^[^ ^43^, ^44^ ^]^ Additionally, it is employed to determine the ecological security status of a given region.^[^ ^45^ ^]^ Furthermore, the index can be utilized for the assessment of regional biodiversity.

(3) $$T=\frac{\mathrm{ef}}{\mathrm{ec}}$$

In the above equations, T is the ecological pressure index (**Table** **3**); ef is the per capita ecological footprint; ec is the per capita ecological carrying capacity of land.

**Table 3:** Security level of land ecological pressure index.

Security Level	Land Ecological Pressure Index	Safe State
I	T<0.5	Safe
II	0.5≤T≤0.8	relatively safe
III	0.8≤T≤1.0	transition state
IV	T>1.0	unsafe

### 2.4 Suitability Evaluation

This study employs the Minimum Impedance Theory and Minimum Cost Result Response (*MCRR*) model to evaluate the suitability of rural settlements in Nangqen County, based on the effect of six factors on the spatial distribution pattern of rural settlements in the region. These factors include population distribution characteristics, land use status, rural settlements dispersion and density. The specific Equation (4) of impedance is as follows:

(4) $$\mathrm{MCRR}=\mathrm{fmin}\sum \left(\mathrm{Di},j\times \mathrm{Ri}\right)$$

In the above equations, *MCRR* represents the Minimum Cost Result Response, which represents the resistance that the substance overcomes when reaching its destination; *f* represents a functional relationship between *MCRR* and (D_i,j_×R_i_); min represents the minimum impedance value; D_i, j_ represents the diffusion distance of the substance from the i‐th unit to the j‐th unit during the process of moving from the “source” to the destination through the i‐th unit; R_i_ is the impedance coefficient of landscape unit i.

In accordance with the tenets of scientificity, comprehensiveness, conciseness, and operability in the construction of the indicator system, and with full consideration of the 3D of ecology, production, and life, the three levels of “ecological safety,” “production guarantee,” and “convenience of life” were identified as the initial selection of indicators, which were based on their relevance to rural land patterns and their suitability for measurement (**Tables** 3 and **4**). Nine indicators were identified as being highly pertinent to rural land patterns and were thus selected for further analysis. These indicators include slope, geological disaster risk, altitude, agricultural and animal husbandry radius, per capita production land area, public service facilities, water source distance, transportation accessibility, and urban radiation. A 1 km x 1 km grid is employed as the fundamental evaluation unit, with the objective of standardizing the data format, data type, spatial reference, and spatial resolution of the aforementioned indicators. The study adopted the AHP‐Entropy Method, a hybrid weighting approach, for indicator weighting. This methodology was selected to diminish the subjective bias inherent in the expert scoring of the Analytic Hierarchy Process (*AHP*) while harnessing the objectivity and scientific merits of the Entropy Method in indicator weighting. The full list of indicators is presented in **Figure** **3** (**Table** **5**).

**Table 4:** List of evaluation indicators.

Target Layer	Criterion Layer	Basic Index Layer/Weight				
		1	2	3	4	5
Ecological Security	Slope	0–3°	3°–8°	8°–15°	15°–25°	>25°
	Geological hazard risk	>2000	2000	1000	500	200
	Elevation	<3000	3500–3700	3700–3900	3900–4100	4100–4300
Production Guarantee	Farming husbandry radius	0–500	500–1000	1000–1500	1500–2000	>2000
	Per capita production land area	>0.87	0.83–0.87	0.75–0.83	0.70–0.75	<0.7
Living Convenience	Public service facilities	<200	200–500	500–1000	1000–2000	>2000
	Water source distance	0–500	500–1000	1000–1500	1500–2000	>2000
	Transportation accessibility	0–500	500–1000	1000–1500	1500–2000	>2000
	Urban radiation	0–500	500–1000	1000–1500	1500–2000	>2000

Figure 3The rural suitability evaluation.

**Figure:**
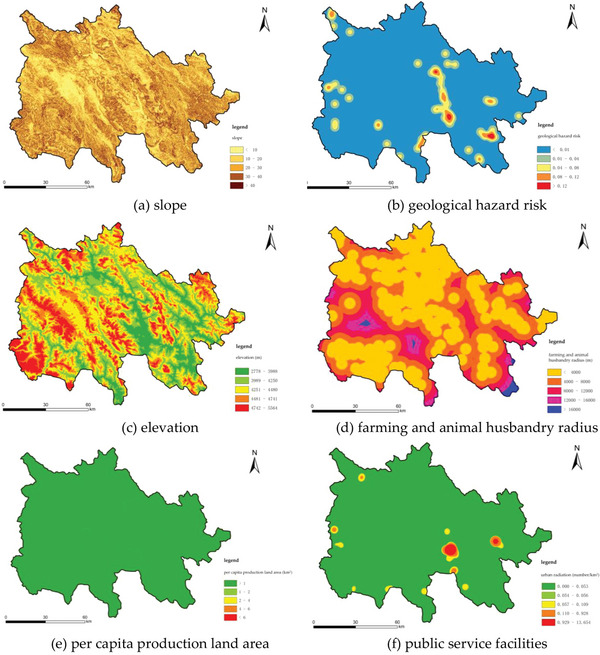

**Figure:**
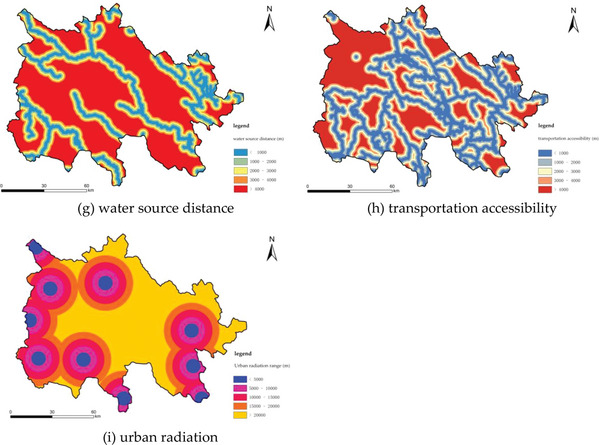

**Table 5:** List of indicator weights.

Factor	Index	AHP Weight	Principal Component Analysis Weight	Average Weight
Ecological security	Slope	0.325	0.106	0.216
	Geological hazard risk	0.157	0.035	0.096
	Elevation	0.125	0.013	0.069
Production guarantee	Farming husbandry radius	0.107	0.060	0.084
	Per capita production land area	0.106	0.173	0.139
Living convenience	Public service facilities	0.071	0.071	0.071
	Water source distance	0.060	0.157	0.109
	Transportation accessibility	0.035	0.153	0.094
	Urban radiation	0.013	0.125	0.069

The rural suitability evaluation model of Nangqen County employs a multi‐factor weighted evaluation approach. The scores and weights assigned to the identified evaluation factors are used to calculate the resistance value of land suitability for rural residential units in Nangqen County. The expression formula is as follows:

(5) $$Z=\sum_{i=1}^{n}F_{\mathrm{ij}}\times W_{j}$$

In the above equations, Z is the comprehensive resistance value of rural residential land patches; F_ij_ represents the score of the j‐th indicator of the i‐th grid unit; W_j_ represents the weight of the j‐th indicator; n is the number of evaluation indicators.

## 3 Results

### 3.1 Changes in Ecological Footprint

The total ecological footprint of Nangqen County was calculated for five periods: 2000, 2005, 2009, 2015, and 2020. The resulting trend is presented in **Table** **6**.

**Table 6:** Total ecological footprint of Nangqen County.

Land Type	2000 [hm^2^]	2005 [hm^2^]	2009 [hm^2^]	2015 [hm^2^]	2020 [hm^2^]
Farmland	14 719.7655	19 495.9000	29 017.4325	45 515.1200	53 687.5115
Forest land	43 912.5324	56 832.7000	106 088.2825	136 716.7200	140 743.4595
Grassland	1778.9970	2303.7600	3973.6975	4645.7600	4984.9464
Waters	120.51270	172.1200	257.4750	323.6800	352.1973
Fossil energy land	114.7740	205.2200	446.2900	656.8800	695.3639
Building land	13 009.6329	16 185.9000	21 516.3275	25 275.6000	26 052.6664
Total	73 656.2145	95 195.6000	161 299.5050	213 133.7600	226 516.1450

**Figure 4:**
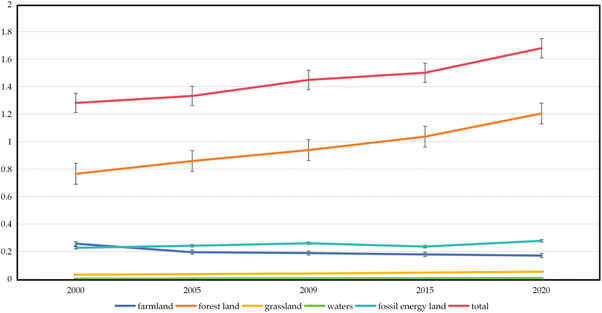
Nangqen County's ecological footprint per capita increased year by year from 2000 to 2020.

The per capita ecological footprint of Nangqen County exhibited a pronounced growth trend from 2000 to 2020, rising from 1.2835 hm^2^ in 2000 to 2.50829 hm^2^ in 2020, representing a more than twofold increase (**Figure** **4**). Among these, the increase in the footprint of grassland is the most significant, with a growth rate of over three times over the past 20 years. This has become the main factor driving the overall increase in the ecological footprint. The footprint of cultivated land has also increased, indicating the expansion of agricultural production activities and the improvement of the quality of life. Concurrently, the ecological footprint of construction land and fossil energy land is also on the rise, reflecting the acceleration of urbanization and the growth of energy demand.

From 2000 to 2020, the per capita ecological footprint of Nangqen County exhibited a notable increase. The proportion of grassland ecological footprint rose from 59.62% to 62.13%, becoming the most significant contributor to the growth (**Table** **7**). This reflects the expansion of animal husbandry activities and the high dependence on grassland resources. The proportion of the ecological footprint of cultivated land has also increased from 19.98% to 23.70%, with a significant increase in per capita area. This indicates a demand for agricultural production expansion and an improvement in the quality of life. Although the changes in the ecological footprint of forest and water areas are relatively small, the continuous increase in the overall ecological footprint reveals that Nangqen County is facing severe challenges to the ecological environment during the social transformation period of diversified consumption, accelerated urbanisation, and increased energy demand. This emphasises the urgency of maintaining ecological balance and efficient utilisation of resources.

**Table 7:** Per capita ecological footprint of Nangqen County.

Land Type	2000 [hm^2^]	2005 [hm^2^]	2009 [hm^2^]	2015 [hm^2^]	2020 [hm^2^]
Farmland	0.2565	0.2945	0.3381	0.4781	0.5945
Forest land	0.7652	0.8585	1.2361	1.4361	1.5585
Grassland	0.0310	0.0348	0.0463	0.0488	0.0552
Waters	0.0021	0.0026	0.0030	0.0034	0.0039
Fossil energy land	0.0020	0.0031	0.0052	0.0069	0.0077
Building land	0.2267	0.2445	0.2507	0.2655	0.2885
Total	1.2835	1.4380	1.8794	2.2388	2.5083

A review of the ecological footprint dynamics of Nangqen County from 2000 to 2020 reveals the following key points:

1. Population growth pressure: The population of Nangqen County has increased by ≈57.36% over the past 20 years, resulting in a concomitant increase in the total ecological footprint. Population growth not only increases basic living needs such as food, housing, and energy, but also intensifies the demand for resources such as farmland and grassland, promoting the expansion of the ecological footprint.
2. Livestock activities: As a region dominated by animal husbandry, the sharp increase in grassland ecological footprint in Nangqen County is closely related to overgrazing and grassland expansion. As a consequence of population growth, the demand for livestock products has increased, leading to an excessive utilization of grassland resources. This has resulted in a deterioration of the ecological balance, manifested as problems such as soil erosion and soil erosion.
3. Economic development and changes in consumption patterns: Over the past 20 years, Nangqen County has experienced rapid economic and social transformation, with significantly improved living standards and diversified consumption patterns, leading to an increase in resource consumption. For example, the increasing demand for fossil fuels and the expansion of construction land are direct manifestations of the acceleration of economic activities and the process of urbanization.
4. Resource management and utilization efficiency: Although the ecological footprint changes of forest and water areas are relatively small, indicating a certain degree of resource utilization stability, overall, insufficient resource management and low utilization efficiency remain the problems. These fail to effectively alleviate the rapid rise of ecological footprint.
5. Natural environmental factors: The unique geographical environment of Nangqen County, such as high cold, low oxygen, and drought, poses challenges to biological adaptability, limiting the ecological carrying capacity itself. Furthermore, the combination of climate change with other factors serves to exacerbate the fragility of the ecological environment.

### 3.2 Changes of Ecological Carrying Capacity

**Figure 5:**
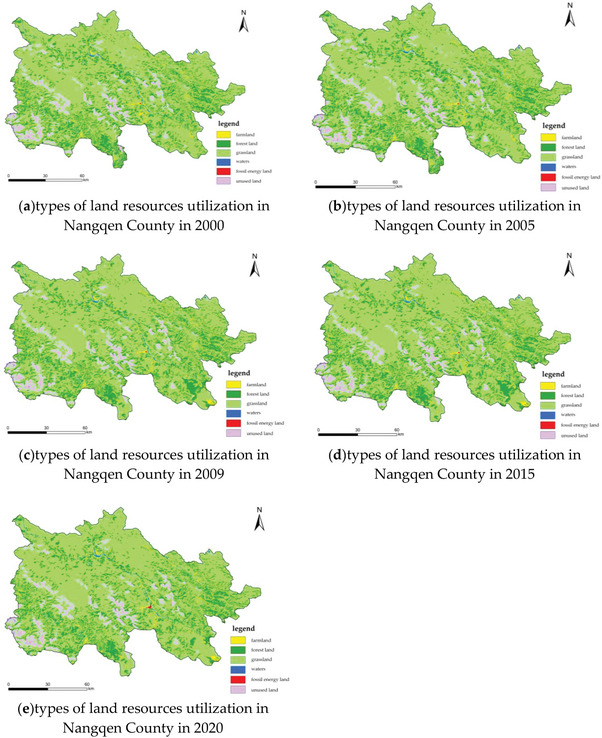
Change of land resource utilization in Nangqen County from 2000 to 2020.

The overall land ecological carrying capacity of Nangqen County declined slowly from 2000 to 2005, the ecological carrying capacity of cultivated land declined from 2000 to 2015, and rose slightly from 2015 to 2020(**Figure** **5**, **Table** **8**). The ecological carrying capacity of grassland and fossil energy land both decreased slowly between 2000 and 2015, and slightly increased between 2015 and 2020. The ecological carrying capacity of forest land remained stable after increasing between 2009 and 2015. The ecological carrying capacity of water bodies fluctuated, and after a brief decline from 2000 to 2005, it showed an upward trend. The ecological carrying capacity of construction land has decreased significantly, from 424.103 hm^2^ in 2000 to 333.695 hm^2^ in 2020, a decrease of 21.32% over the past 20 years. The trend of changes in the ecological carrying capacity of various types of land is complex, but overall it shows a downward trend.

**Table 8:** Total ecological footprint of Nangqen County.

Land Type	2000 [hm^2^]	2005 [hm^2^]	2009 [hm^2^]	2015 [hm^2^]	2020 [hm^2^]
Farmland	6643.8537	6270.6759	6323.0367	6011.7658	6178.0102
Forest land	73 957.0601	95 979.0902	158 996.5799	198 601.6476	207 306.2567
Grassland	7772.0362	9297.6576	9901.4586	16 235.7888	17 213.2367
Waters	7304.4010	8004.8404	8193.0605	7876.2844	8046.1947
Fossil energy land	2284.6453	2635.5014	1708.3981	1895.0131	1797.6150
Building land	0	0	0	0	0
Total	97 961.9963	122 187.7655	185 122.5338	230 620.4997	240 541.3133

It can be seen from **Table** **9** that the per capita land ecological carrying capacity of Nangqen County decreased significantly from 10.09845408 hm^2^ in 2000 to 6.79373464 hm^2^ in 2013, with an average annual decrease of 0.367191049 hm^2^; The decline rates of land ecological carrying capacity are 13.29%, 22.41%, 9.43%, and ‐4.89%, respectively, with an average annual decrease rate of 2.01%. The per capita ecological carrying capacity of cultivated land fluctuates slightly, showing a decline from 2000 to 2015, and has rebounded since 2015 to 2020. Its share in the total per capita ecological carrying capacity fluctuates slightly, but overall shows a downward trend. The per capita ecological carrying capacity of grassland has shown a significant downward trend, from 6.6266816 hm^2^ in 2000 dropped to 4.3122156 hm^2^ in 2020, It has decreased by ≈ 34.93% in 20 years. Grassland is the largest provider of land ecological carrying capacity in the study area, accounting for 65.62%, 65.66%, 66.70%, 66.44%, and 66.82% of the per capita land ecological carrying capacity in 20 years, respectively.

**Table 9:** Per capita ecological carrying capacity of Nangqen County.

Land Type	2000 [hm^2^]	2005 [hm^2^]	2009 [hm^2^]	2015 [hm^2^]	2020 [hm^2^]
Farmland	0.1158	0.0947	0.0737	0.0631	0.0684
Forest land	1.2887	1.4498	1.8526	2.0862	2.2956
Grassland	0.1354	0.1404	0.1154	0.1705	0.1906
Waters	0.1273	0.1209	0.0955	0.0827	0.0891
Fossil energy land	0.0398	0.0398	0.0199	0.0199	0.0199
Building land	0	0	0	0	0
Total	1.7070	1.8456	2.1571	2.4224	2.6636

The change of grassland cover directly controls the lifeblood of land ecological carrying capacity in Nangqen County. The per capita ecological carrying capacity of forest and water areas is gradually decreasing. Due to the reduction of grassland area, the ecological carrying capacity of fossil energy land has been decreasing year by year. Although there are significant changes in the ecological carrying capacity of various types of land, the proportion change in the overall composition is not significant, maintaining a stable structure, which also means that the carrying capacity of all types of land shows varying degrees of degradation.

### 3.3 Changes of Ecological Pressure

In the initial stage, the ecological footprint was clearly in surplus, as evidenced by the 2000 figure of 1.2835 hm^2^, which was below the per capita ecological carrying capacity of 1.7070 hm^2^. This surplus reached 0.4235 hm^2^. Nevertheless, over time, despite the continuous growth of ecological carrying capacity and the faster growth rate of per capita ecological footprint, the ecological surplus has been shrinking year by year. By 2020, although the per capita ecological carrying capacity had increased to 2.6636 hm^2^, the surplus was only 0.1553 hm^2^, given a per capita ecological footprint of 2.5083 hm^2^. This trend reflects the fact that with economic development and increasing social activity, the demand for natural resources has increased significantly, placing greater pressure on the ecosystem (**Table** **10**).

**Table 10:** Per capita ecological carrying capacity of Nangqen County.

Year	Per Capita Ecological Footprint [hm^2^]	Per Capita Ecological Carrying Capacity [hm^2^]	Ecological Surplus [hm^2^]
2000	1.2835	1.7070	0.4235
2005	1.4380	1.8457	0.4077
2009	1.8794	2.1570	0.2776
2015	2.2388	2.4225	0.1837
2020	2.5083	2.6636	0.1553

**Figure 6:**
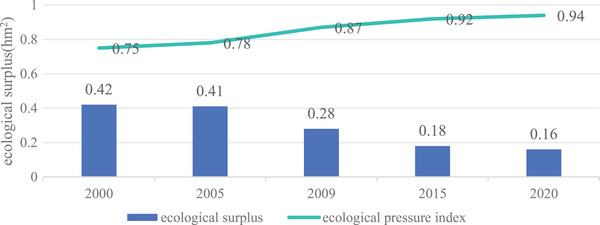
Change of land resource utilization in Nangqen County from 2000 to 2020.

Research indicates that the land ecology in Nangqen County remained relatively stable from 2000 to 2005, while the ecological pressure index exhibited a deteriorating trend from 2005 to 2020, suggesting a decline in ecological environment security. As a key indicator for evaluating land planning, the ecological pressure index highlights the urgent problem of ecological footprint expansion and carrying capacity reduction in Nangqen County. This indicates severe ecological challenges in the future. Despite the implementation of a series of ecological protection measures, land resources continue to face a continuous increase in pressure(**Figure** **6**). This may be due to population growth, the expansion of economic development activities, and an increased intensity of resource development and utilization.

### 3.4 Suitability Evaluation

**Figure 7:**
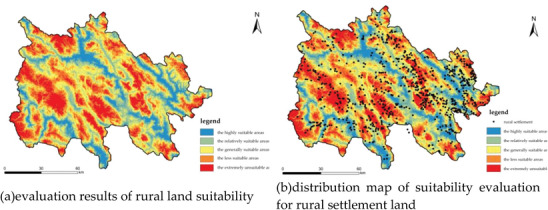
By evaluating the suitability evaluation system for rural settlements established earlier, a) is obtained, while b) is obtained based on the actual location of rural settlements.

Using Arc GIS Spatial Analysis Tool and multi factor comprehensive evaluation model, the suitability of rural settlements in Nangqen County was comprehensively evaluated, and the comprehensive resistance range of rural settlement land patches in Nangqen County was 1.001‐3.904. The lower the comprehensive resistance score, the higher the suitability. In order to clearly express and analyze the spatial differentiation of ecological carrying capacity and various dimensions of carrying capacity, the suitability of rural residential space is divided into five levels using the natural discontinuity method. These levels are defined as follows: most suitable, more suitable, generally suitable, unsuitable, and unsuitable (**Figure** **7**).Overlay the current plot data of rural settlement land in Nangqen County with the suitability evaluation zoning result map to calculate the area, quantity and proportion of rural settlement land with different suitability in Nangqen County. Combined with the location data of rural settlements in Nangqên County, the suitability evaluation results of all villages can be obtained.

It can be seen from **Table** **11** that the generally suitable areas of Nangqen County is 401.8509km^2^ at the highest, accounting for 26.56% of the total land area of Nangqen County. The number of patches in this area is 446 501 at the highest. The highly suitable areas are mainly distributed in the central area of Nangqen County and the surrounding areas of various towns, the traffic in this area is convenient. The area of highly suitable areas and relatively suitable areas is 178.1892 km^2^ and 329.2407 km^2^ respectively, accounting for 11.78% and 21.76% of the total area of Nangqen County, 33.54% in total. The area of the less suitable areas and extremely unsuitable areas in Nangqen County is 374.41172 and 229.5441km^2^ respectively, accounting for 24.74% and 15.17% of the total area, accounting for 39.91% in total. This area has high altitude, insufficient arable land resources and fragile ecological environment, so it is necessary to optimize the land use of rural settlements in Nangqen County.

**Table 11:** Statistical table of land suitability evaluation for rural residential units in Nangqen County.

Suitability Level	Area [km^2^]	Number of Patches	Proportion	Number of Villages
The highly suitable areas	178.1892	197 988	11.78%	84
The relatively suitable areas	329.2407	365 823	21.76%	140
The generally suitable areas	401.8509	446 501	26.56%	233
the less suitable areas	374.4117	416 013	24.74%	207
the extremely unsuitable areas	229.5441	255 049	15.17%	147

## 4 Discussion

Although the agricultural and pastoral regions in Nangqen County cover extensive areas, most are not conducive to human habitation. Additionally, the harsh climate and environmental conditions restrict crop growth, hindering the development of agricultural production. According to statistical data, the per capita grassland area in Nangqen County is 11.79 hm^2^, significantly exceeding the national average of 0.33 hm^2^.^[^ ^46^ ^]^ Herdsmen view grasslands, similar to agricultural land, as the primary material source for their livelihoods. However, the grasslands on the Qinghai‐Tibet Plateau differ from cultivated land due to their high altitude, high degradation, and difficulty in determining ownership, posing challenges for collective management. The absence of effective regulatory mechanisms means that traditional grazing practices can lead to severe damage to grasslands, causing a significant depletion of grassland resources. This results in a decrease in supply and an increase in demand, further intensifying the pressure on grasslands. Among all types of land in Nangqen County, grassland experiences the most rapid decline in its ecological carrying capacity. This decline is evidenced by a decrease in the area of grassland from 6.6267 hm^2^ in 2000 to 4.3122 hm^2^ in 2020. Furthermore, the average annual decrease rate is 11.57%. The reduction in per capita grassland directly leads to a decrease in per capita ecological carrying capacity. Factors such as overgrazing, grassland degradation, and the diminished ecological restoration function contribute to the reduction of grassland area.

Furthermore, the increasing population and the growing demand for land are also significant reasons for the escalating ecological pressure on grasslands. When comparing the total land ecological carrying capacity in 2000 and 2020, it is evident that notable changes have occurred in the ecological carrying capacity of arable land. However, the overall trend of total land ecological carrying capacity change is not as apparent as the trend of per capita land ecological carrying capacity change, which shows a distinct downward trajectory. The primary reason for this discrepancy is that the latter calculation process takes into account the impact of population changes on ecological carrying capacity. Nangqen County's permanent population increased from 57387 in 2000 to 90307 in 2020, with an average annual growth rate of 1.82%. As the population has grown, the average amount of land per person has decreased, which has indirectly contributed to the rapid decline in the per capita ecological carrying capacity of land.

In addressing the issue of land ecological carrying capacity in Nangqen County, it is essential to adhere to the principle that ecological protection and economic development are mutually reinforcing, transcending the dichotomy between the two. On the one hand, economic development must be founded on land ecological carrying capacity. On the other hand, ecological protection restricts the extensive economic development model, without hindering the healthy growth of the economy. The future development of Nangqen County must be based on the overarching premise of an eco‐tourism city, prioritizing ecology and driving economic benefits through ecological production capacity. It is crucial to cultivate residents' correct ecological perspectives and achieve green utilization of land resources. At the government level, comprehensive ecological compensation must be strengthened from all angles, protecting residents' legitimate rights and interests, and developing and utilizing this precious land of the Sanjiangyuan region in a systematic and reasonable manner.

The research outcomes of the suitability evaluation of rural settlements reveal that the distribution of rural settlements in Nangqen County is not rational. With the support of urban and rural planning and landscape ecology methods, and through the analysis of various data on rural settlements in Nangqen County, the following issues within the rural settlements have been identified: small scale, scattered spatial layout, significant fragmentation, and unstable spatial morphology. The socio‐economic and natural conditions in the upper reaches of the Lancang River are complex and diverse. The coordination of the overall development of rural settlements with the protection of the ecological environment in the region is a critical issue. The dialectical relationship between the two stems from the perpetual conflict between “development and protection,” as well as the intricate interplay of factors such as the fragmented spatial status of rural settlements in the upper reaches of the Lancang River, backward economic development, and a fragile natural environment.

A review of current data and the adaptability of rural settlements has revealed that highly suitable and relatively suitable villages offer a high degree of production security, convenient living conditions, and a favorable ecological environment, with high agglomeration benefits. A total of 227 such villages have been identified, with the majority located in the east‐central area of Nangqen County. These villages possess advantageous locations, convenient transportation, good living conditions, and well‐developed social infrastructure. In comparison to higher‐level planning, it is evident that this is the primary direction of regional development. In order to optimize this type of rural settlement, the government can guide the scattered, small‐scale settlements in the surrounding areas to migrate to such settlements, gradually forming larger rural settlements. This approach will improve the level of public infrastructure construction and intensify agriculture and animal husbandry, leveraging their unique geographical advantages and vigorously tapping into the development potential of their cultural tourism industry. The establishment of a green, ecological, and environmentally friendly characteristic rural tourism industry can be achieved through the development of religious tourism, leisure, health and wellness, ecological education, and other services and distinctive tourism products. This will result in the improvement of the basic supporting service facilities of rural tourism, the achievement of a scientific layout within the settlement, and the promotion of local economic development.

**Figure 8:**
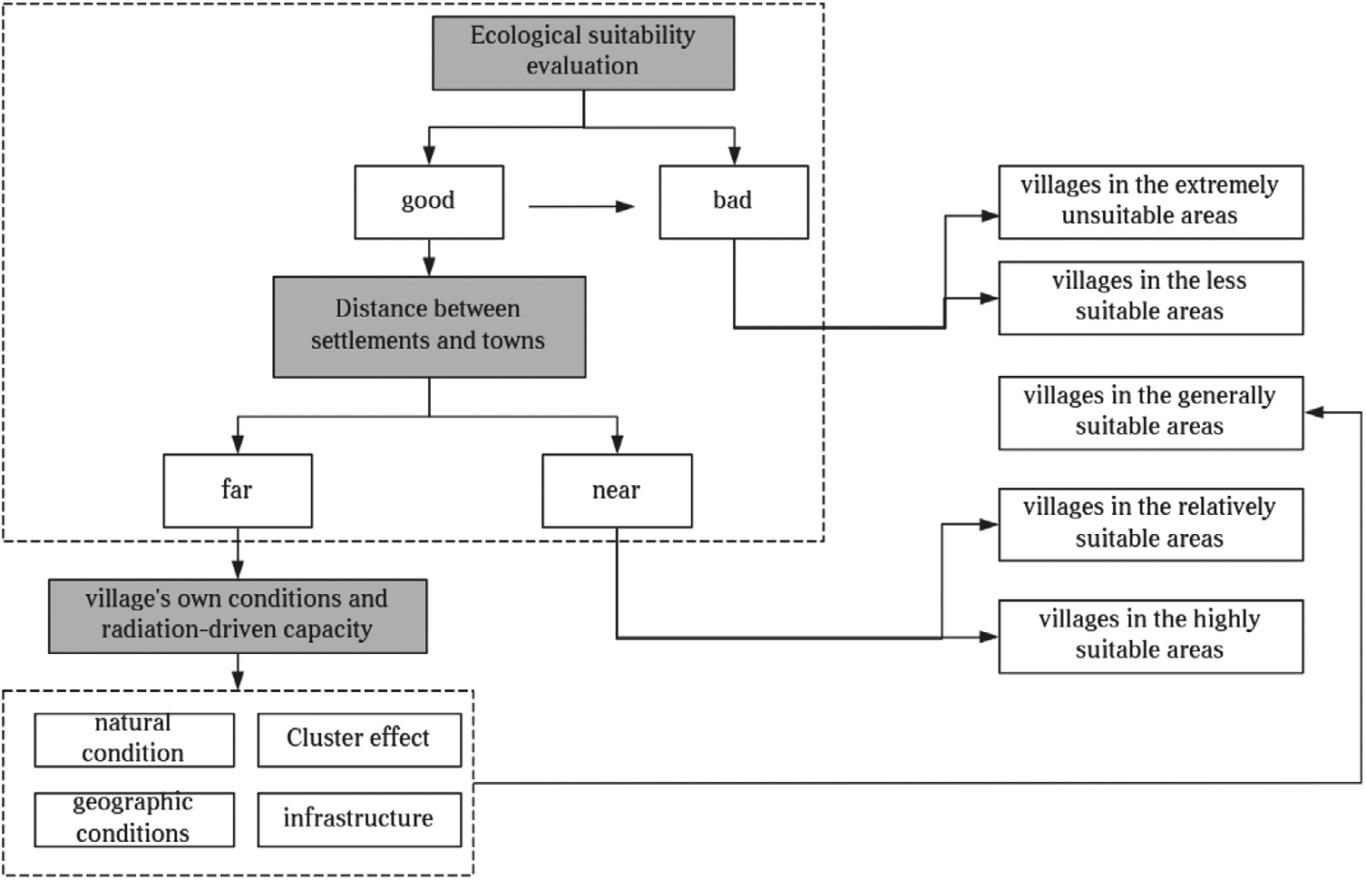
Analysis framework and method of differentiated settlement space improvement.

The results of the ecological suitability assessment of rural settlements inNangqen County demonstrate notable disparities between different settlements. Consequently, it is imperative to devise and implement differentiated spatial improvement strategies (**Figure** **8**, Table 11). In the case of villages deemed to be “highly suitable” or “relatively suitable”, the objective should be to safeguard and enhance the existing ecological and socio‐economic advantages. This encompasses the implementation of green conservation measures, including forest restoration and biodiversity conservation, as well as the moderately improved infrastructure of clean energy promotion and enhanced waste management systems. These measures can consolidate the sustainable development foundation of existing settlements and serve as exemplars for rural development, influencing and guiding the surrounding areas to achieve green transformation.

For villages deemed to be generally suitable, the improvement strategy should prioritize the dual enhancement of the ecological and socio‐economic aspects. In particular, the following measures should be implemented: optimization of water resource management, adoption of water‐saving irrigation and rainwater collection technologies, and improvement of water use efficiency; introduction of modern agricultural technology to enhance agricultural output and alleviate pressure on natural resources. Furthermore, the enhancement of educational and healthcare services, such as the establishment of distance learning centres and mobile medical stations, can lead to an improvement in the quality of life for residents. From an economic perspective, the exploration of green industries, such as ecotourism and the processing of specialty agricultural products, can facilitate the creation of employment opportunities and enhance the economic self‐sufficiency of villages.

The strategies of ecological migration and settlement integration are regarded as reasonable and necessary for the development of villages deemed to be “less suitable” and “very unsuitable” due to their extremely fragile ecological environments and high incidence of natural disasters. This process requires meticulous planning, with due consideration of the desires and well‐being of residents, respect for local culture and traditions, and assurance of a seamless transition of the social economy. The establishment of an ecological compensation mechanism to provide employment training and entrepreneurial funds for relocated residents effectively mitigates uncertainty during the migration process. Concurrently, the planning of the relocation site should contemplate ecological carrying capacity, eschew excessive development, and achieve economic and ecological harmony through novel urbanization models such as smart villages (**Table** **12**).

**Table 12:** Development strategy of settlement classification.

Settlement Type Ecological Security Status	Ecological Surplus Area	Ecologically Balanced Area	Ecological Deficit Area
Villages in the highly suitable areas	Develop eco‐tourism, protect natural resources, establish ecological compensation mechanism, and promote green buildings and technology.	Strengthen the education of ecological protection awareness, moderately develop ecological agriculture, and control population growth.	Restrict development activities, implement environmental remediation projects, and guide population migration to other suitable areas.
Villages in the relatively suitable areas	On the basis of protecting the existing ecological resources, moderately develop industries that are compatible with the environment.	Promote community participatory management, carry out ecological restoration projects, and optimize the industrial structure.	Implement strict environmental protection measures, reduce pollutant emissions, and strengthen ecological monitoring.
Villages in the generally suitable areas	Establish and improve environmental protection laws and regulations, encourage the use of renewable energy, and enhance residents' awareness of environmental protection.	Balance the relationship between economic development and environmental protection, make rational use of land resources, and enhance the self‐management ability of communities.	Reduce dependence on natural resources, promote green transformation, and provide technical support for ecological restoration.
Villages in the less suitable areas	Strictly restrict further expansion of human activities and promote ecological migration policy.	We will implement strict environmental protection policies and gradually improve the quality of the ecological environment.	Focus on the treatment of pollution sources, implement the emergency ecological restoration plan, and consider the problem of population resettlement.
Villages in the extremely unsuitable areas	Enforce ecological migration and prohibit all destructive human activities.	For some areas, we can consider setting up nature reserves to restrict human access.	Concentrate resources for ecological reconstruction, and take government led ecological relocation measures when necessary.

## 5 Conclusion

This study provides a systematic evaluation of the rural human settlement environment in the upper reaches of the Lancang River, specifically in Nangqen County, within the constraints of ecological carrying capacity. The primary objective was to determine whether the production and consumption behaviors of local residents were sustainable within the region's ecological limits. Through the application of the ecological footprint model combined with ArcGIS platform analysis, we calculated the ecological footprint, ecological carrying capacity, and ecological deficit of Nangqen County from 2000 to 2020. The results revealed several key findings.

First, the total ecological footprint of Nangqen County more than doubled over the past 20 years, primarily driven by the expansion of grassland usage for livestock activities. This indicates a significant increase in resource consumption, particularly in relation to animal husbandry. Second, despite the fluctuation in ecological carrying capacity, it remained relatively stable, although the per capita carrying capacity declined significantly due to population growth. This suggests that the region's ecological system is facing increasing pressure. Third, the land ecological evaluation results showed a high degree of coincidence with the location of towns and transportation networks, implying that human activities are concentrated in areas with relatively higher ecological carrying capacity.

Furthermore, the suitability evaluation of rural settlements in Nangqen County revealed that ≈43.4% of the settlements are located in areas deemed relatively unsuitable or extremely unsuitable for human habitation. This highlights the need for optimization of settlement patterns to promote sustainable development. The study also identified several strategies for improving rural settlements, including eco‐tourism development in highly suitable areas, ecological migration in unsuitable areas, and balanced economic and ecological development in generally suitable areas.

Despite these contributions, the study has several limitations. First, the research scope is limited to Nangqen County, and broader regional comparisons could provide more insight. Second, the determination of ecological thresholds requires further validation and testing. Lastly, the study focuses mainly on quantitative analysis, and qualitative research on local socio‐economic conditions and resident behavior patterns could enrich the understanding.

In conclusion, this study underscores the importance of considering ecological carrying capacity in rural development planning. It emphasizes the need for adaptive strategies to optimize rural settlements and ensure sustainable utilization of ecological resources. Future research should expand the geographical scope, deepen the exploration of ecological thresholds, and integrate qualitative and quantitative methods to provide a more comprehensive understanding of rural human settlement environments within ecological constraints.

## Conflict of Interest

The authors declare no conflict of interest.

## Data Availability

The data that support the findings of this study are openly available in Geospatial Data Cloud at https://www.gscloud.cn/sources/accessdata/421?pid=302.

## References

[1] Q. Wu , J. Zhu , X. Zhao , China. Forests 2023, 14, 234.

[2] A. Smailes , M. Chisholm , The Geograph. J. 1962, 128, 354.

[3] B. FU , Z. OUYANG , P. SHI , J. FAN , X. WANG , H. ZHENG , W. ZHAO , F. WU , Bull. Chin. Acad. Sci. (Chinese Version) 2021, 36, 1298.

[4] J. C. Hudson , Annals Associat. American Geograph. 1969, 59, 365.

[5] Y. Hu , T. Wu , L. Guo , S. Zhang , Land 2023, 12, 1635.

[6] N. Cui , T. Wu , Y.‐C. Wang , H. Zou , J. C. Axmacher , W. Sang , L. Guo , Landscape Ecol. 2022, 37, 1559.

[7] R. Yang , Q. Xu , H. Long , J. Rural Studies 2016, 47, 413.

[8] J. Venetoulis , J. Talberth , Sustainable Development, CRC Press, Boca Raton, Florida 2010, pp. 83–120.

[9] W. E. Rees , The Ecologist 1990, 20, 18.

[10] Z. Zhicong , W. Pei , Landscape Architect. 2021, 28, 117.

[11] Y. Kang , C. Chen , Case Investigation on the Construction Mode of Rural Landscapes in the Qinghai Tibet Plateau, Mengda National Nature Reserve, Heliyon 2024. 10.1016/j.heliyon.2024.e37033 PMC11408023 39296138

[12] T. W. Gillespie , A. Madson , C. F. Cusack , Y. Xue , Arctic, Antarctic, Alpine Res. 2019, 51, 428.

[13] Y. Jiang , Y. Shi , R. Li , L. Guo , Sustainability 2021, 13, 10598.

[14] T. Ma , B. Swallow , J. M. Foggin , L. Zhong , W. Sang , Human. Social Sci. Commun. 2023, 10, 321.

[15] T. Ma , B. Swallow , J. M. Foggin , L. Zhong , W. Sang , Front. Conserv. Sci. 2023, 4, 903788.

[16] H. Xu , T. Zhang , B. Ge , Y. Song , J. Asian Architect. Building Eng. 2023, 22, 2839.

[17] T. Li , R. K. Singh , R. Pandey , H. Liu , L. Cui , Z. Xu , A. Xia , F. Wang , L. Tang , W. Wu , Ecological Indicators 2023, 156, 111134.

[18] Z. Jiapei , X. Xingliang , L. Tong , L. Yali , Y. Yaqian , C. Xiaoyong , J. Res. Ecol. 2022, 13, 955.

[19] X. Wang , D. Zhou , G. Jiang , C. Peng , Front. Environ. Sci. 2023, 11, 1134136.

[20] J. Ptackova , Pastoralism: Research, Policy and Practice 2011, 1, 4.

[21] H. Yang , Y. Xu , K. Zhou , L. Wang , L. Xu , J. Geograph. Sci. 2024, 34, 41.

[22] S. Wu , J. Yan , L. Yang , X. Cheng , Y. Wu , China. Climatic Change 2021, 165, 69.

[23] C. Fang , J. Geograph. Sci. 2023, 33, 639.

[24] T. Li , S. Cai , R. K. Singh , L. Cui , F. Fava , L. Tang , Z. Xu , C. Li , X. Cui , J. Du , Sci. Total Environ. 2022, 838, 155960. 35588815 10.1016/j.scitotenv.2022.155960

[25] M. Wackernagel , L. Onisto , P. Bello , A. C. Linares , I. S. L. Falfán , J. M. García , A. I. S. Guerrero , M. G. S. Guerrero , Ecol. Econom. 1999, 29, 375.

[26] Y. Wang , Social and Economic Stimulating Development Strategies for China's Ethnic Minority Areas, Springer, New York 2023, pp. 377–391.

[27] X. Duan , Y. Chen , L. Wang , G. Zheng , T. Liang , J. Environ. Manage. 2023, 325, 116539. 36274338 10.1016/j.jenvman.2022.116539

[28] Z. Binbin , C. Yang , L. Yuhan , E3S Web of Conf. 2019, 136, 03028.

[29] Y. Zhang , D. Li , H. Wang , Q. Xiao , X. Liu , Chin. Sci. Bull. 2006, 51, 1245.

[30] J. Wei , Y.‐M. Guo , L. Sun , T. Jiang , X.‐P. Tian , G.‐D. Sun , Chin. J. Ecol. 2015, 34, 1968.

[31] C. Song , L. Liu , C. Xian , F. Feng , Z. Ouyang , Atmosphere 2023, 14, 1800.

[32] S. Ma , S. Ma , Architectur. Res. 2015, 17, 147.

[33] F. Du , Nomadic Peoples 2012, 16, 116.

[34] E. Cencetti , On the Fringes of the Harmonious Society: Tibetans and Uyghurs in Socialist China 2014, 159.

[35] Z. Wang , K. Song , L. Hu , Ambio 2011, 40, 102.

[36] D. Chu , Y. Zhang , C. Bianba , L. Liu , J. Geograph. Sci. 2010, 20, 899.

[37] Y. Shen , G. Wang , G. Wang , J. Pu , X. Wang , Sci. Cold Arid Regions 2009, 1, 0475.

[38] J. Zhang , L. Zhang , X. Liu , Q. Qiao , Sustainability 2019, 11, 4659.

[39] V. Niccolucci , M. Panzieri , E. Tiezzi , WIT Trans. Ecol. Environ. 1970, 46.

[40] G. A. Cornia , World Dev. 1985, 13, 513.

[41] W. R. Catton , Overshoot: The ecological basis of revolutionary change, University of Illinois Press, Champaign 1982.

[42] A. Galli , T. Wiedmann , E. Ercin , D. Knoblauch , B. Ewing , S. Giljum , Ecol. Indicators 2012, 16, 100.

[43] J. D. Bastedo , J. G. Nelson , J. B. Theberge , Environ. Manage. 1984, 8, 125.

[44] G. W. Guo Wen , S. T. Sun Tao , G. M. Gao MingMei , Environ. Sci. Technol. 2013, 36, 172.

[45] B. Ness , E. Urbel‐Piirsalu , S. Anderberg , L. Olsson , Ecol. Econom. 2007, 60, 498.

[46] Y. Jiang , B. Li , Y. Yuan , J. Global Change Data & Discov. 2020, 2, 163.

